# Design of Mixed Medicinal Plants, Rich in Polyphenols, Vitamins B, and Palmitoylethanolamide-Based Supplement to Help Reduce Nerve Pain: A Preclinical Study

**DOI:** 10.3390/ijms25094790

**Published:** 2024-04-27

**Authors:** Simone Mulè, Giorgia Rosso, Mattia Botta, Arianna Brovero, Sara Ferrari, Rebecca Galla, Claudio Molinari, Francesca Uberti

**Affiliations:** 1 Department for Sustainable Development and Ecological Transition, University of Piemonte Orientale (UPO), 13100 Vercelli, Italy; simone.mule@uniupo.it (S.M.); rossog89@gmail.com (G.R.); mattiabotta23@gmail.com (M.B.); sara.ferrari@uniupo.it (S.F.); rebecca.galla@uniupo.it (R.G.); claudio.molinari@uniupo.it (C.M.); 2 Department of Clinical and Biological Sciences, University of Turin, 10043 Turin, Italy; arianna.brovero@unito.it; 3 Noivita Srls, Spin Off, University of Piemonte Orientale (UPO), Via Solaroli 17, 28100 Novara, Italy

**Keywords:** natural extracts, neuropathy, nutraceutical approach, nerve injury, intestinal absorption, synergy effect

## Abstract

Neuropathy affects 7–10% of the general population and is caused by a lesion or disease of the somatosensory system. The limitations of current therapies highlight the necessity of a new innovative approach to treating neuropathic pain (NP) based on the close correlation between oxidative stress, inflammatory process, and antioxidant action. The advantageous outcomes of a novel combination composed of Hop extract, Propolis, Ginkgo Biloba, Vitamin B, and palmitoylethanolamide (PEA) used as a treatment was evaluated in this study. To assess the absorption and biodistribution of the combination, its bioavailability was first examined in a 3D intestinal barrier model that replicated intestinal absorption. Further, a 3D nerve tissue model was developed to study the biological impacts of the combination during the essential pathways involved in NP. Our findings show that the combination could cross the intestinal barrier and reach the peripheral nervous system, where it modulates the oxidative stress, inflammation levels, and myelination mechanism (increased NRG, MPZ, ERB, and p75 levels) under Schwann cells damaging. This study proves the effectiveness of Ginkgo Biloba, Propolis, Hop extract, Vitamin B, and PEA in avoiding nerve damage and suggests a potential alternative nutraceutical treatment for NP and neuropathies.

## 1. Introduction

Pain is an unpleasant emotional and sensory experience that causes (or may cause) tissue damage [1]. It is an acute signal for tissue damage transmitted from the brain’s periphery by certain receptors and fiber systems. The immediate result of these processes is loss or diminution of function, which includes discomfort.

Chronic pain, defined as pain lasting three months or more, is the main cause of disability globally when all pain syndromes are considered, including low back pain, headache disorders, and neck pain, among others.

Neuropathic pain (NP) is a particularly severe form of chronic pain, arising as a direct consequence of a lesion or disease affecting the somatosensory nervous system [2]. As a direct result of an injury or illness affecting the somatosensory nervous system, NP is an especially severe type of chronic pain [3]. Thus, the coupling of pain and sensory loss, with or without sensory hypersensitivity occurrences in the painful area, is a key characteristic shared by most NP types [4]. From a clinician’s standpoint, it is crucial to differentiate NP from other types of pain that result from real or potential harm to non-neural peripheral tissue. NP typically does not respond well to pain relievers like nonsteroidal anti-inflammatory medicines or opioids, with these medications only providing significant pain relief to less than half of the individuals treated [5]. The prevalence of NP in the general population is estimated to be 7–10%, and it increases to approximately 20–30% in those with diabetes. Prior research has also indicated a higher occurrence of NP, similar to chronic pain in general, in elderly individuals, females, and individuals residing in socially disadvantaged areas [5]. In addition, according to the study context, diagnostic technique, and time of the study, the prevalence is currently estimated to be 1–3%. In contrast, it is 7% among older people [6]. NP is a condition linked to a wide range of illnesses that vary in both their anatomical localisation and underlying cause [7]. Indeed, diseases or lesions affecting the somatosensory nervous system, either centrally or peripherally, may lead to NP. Some examples can be found in painful polyneuropathy, postherpetic neuralgia, trigeminal neuralgia, tinnitus [8], and pain following a stroke. When exposed to either toxic or non-noxious stimuli, NP is clinically characterised by induced heightened pain responses and spontaneous continuous or shooting pain [9]. NP is frequently described as a sensation resembling freezing, squeezing, shooting, pricking, pins and needles, or searing; however, any pain characterisation may be pertinent [10]. Sometimes, in addition to ongoing pain or alone, intermittent electric shock-like pain paroxysms dominate the spontaneous sorrow [11]. Different pathophysiological mechanisms are becoming more identifiable thanks to basic research, and clinical evaluation of symptoms and indicators can assist in identifying the mechanisms responsible for certain NP syndromes [9]. For instance, it has been noted that peripheral neuropathies are, biologically speaking, the clinical expression of malfunctions in the Schwann cells that encase peripheral axons and/or axons themselves [12]. There has also been a growing correlation between peripheral neuropathies and oxidative stress. The most prevalent type of harmful free radicals is reactive oxygen species (ROS), as these compounds have the potential to start molecular instability chain reactions, leading to lipidic peroxidation for example. Oxidative stress is caused by increased oxidant formation, decreased antioxidant defence, or failure to repair oxidative damage [13]. When unbalanced, ROS destroy several important molecules, including enzymes, membranes, lipids, and DNA. Under normal conditions, superoxide dismutase (SOD), catalase, or glutathione, as well as antioxidant Vitamins C and E, remove ROS from cells [14]. Over the years, more and more animal models have been created to investigate the mechanisms behind peripheral neuropathies and correlated diseases like tinnitus or trigeminal neuralgia. However, despite these theories about their development, their aetiology is not well characterised, but the hypothesis that is becoming increasingly popular concerning their development is related to oxidative stress. For instance, the idea that tinnitus is characterised by elevated oxidative stress was developed after a study connected this kind of neuropathy to greater levels of free radicals and plasma scavenger activity [15]. As a result, maintaining this homeostatic condition is critical. Indeed, ROS are required for the body’s functioning, particularly for signal transmission within cells, and the body’s natural antioxidants, such as glutathione S-transferase, catalase, and glutathione peroxidase (GPX), can help regulate their activity [16]. The identification of these phenomena may lead to novel biological options for treating peripheral neuropathies. This is especially significant given that no effective therapeutic method exists despite breakthroughs in reconstructive microsurgery, tissue engineering, regenerative medicine, and a better understanding of peripheral nerve injury pathophysiology [17]. This is why several natural antioxidant therapies are becoming increasingly popular as novel methods for treating NP. Antioxidants can have protective and preventative benefits by limiting the production of free radicals in the body, which would otherwise cause damage to neurons as evidenced by several studies [18]. Considering herbal medicines and their natural components have fewer side effects and fewer difficulties than synthetic treatments, people have been using them more and more in recent years [19]. Globally, there has been a surge in phytopharmaceutical research and the application of medicinal plants and associated substances to treat painful neuropathy [20]. Accordingly, it has been noted that polyphenols with potent antioxidant and anti-inflammatory qualities include resveratrol, bergamot, hydroxytyrosol, and oleuropein [21,22]. Furthermore, in several animal models, these compounds can directly or indirectly activate sirtuins [23]. Thus, the activation of polyphenol-induced sirtuins and glutamatergic transmission modification may represent novel approaches for treating chronic pain [21]. In this context, Ginkgo Biloba is the product that has perhaps received the most testing in preclinical and clinical research about potential novel therapies for neuropathies.

Gingko Biloba has been demonstrated to attenuate mechanical and cold allodynia in a rat model of neuropathic pain, suggesting its use in managing neuropathic pain in humans [24]. In addition, other studies explored the effects of Gingko biloba on spinal cord neuron deterioration in several studies in vivo [25,26]. Finally, it is known to have effects on neuronal plasticity [27], leading to neuroprotection through anti-apoptotic activity [28] that, combined with its antioxidant effect [29,30], improved nerve repair. Indeed, scientific investigations establish that Ginkgo Biloba activates the nuclear factor erythroid 2-related factor 2 (Nrf2) signaling pathway, a crucial molecular mechanism implicated in safeguarding against oxidative stress. The deletion of its gene expression has been found to impede neural regeneration [31]. Regarding the neuropathy aspect, other possible nutraceuticals include Hop extract which can be mentioned because it contains numerous prenylated aromati. These compounds have antioxidant properties that can act against ROS and prevent oxidative damage to proteins and DNA [32]. Hop extract’s potential to lessen oxidative stress may open new application opportunities for the plant as biological research advances. This is remarkably accurate within the field of peripheral neuropathies because it has antinociceptive profiles leading to an opioid system in an in vivo model, supporting its ability to modulate the GABA system [33] involved in pain. Along with the Gingko Biloba and hops described above, B vitamins and palmitoylethanolamide (PEA) may be effective options. Indeed, several studies have shown that patients with peripheral neuropathies suffer from a deficiency of antioxidant B vitamins [13]. Indeed, Vitamin B12 has been suggested as a treatment for NP as it enhances myelination, boosts nerve regeneration, and reduces ectopic nerve firing, among other processes [34]. Furthermore, a study showed that in several patients, less Vitamin B2 intake was significantly associated with neuropathies like tinnitus [35]. Regarding PEA, it has been proposed that PEA functions as a protective endogenous mediator, generated on demand to counteract inflammation, pain, and neuronal damage in inflammatory and neurodegenerative diseases [36]. Furthermore, it has been extensively shown that combining antioxidant molecules with PEA’s anti-inflammatory and protective properties might enhance the compound’s pharmacological effects [36]. Finally, propolis has also gained interest in the field of neuropathic pain as it has been proven to alleviate symptoms of neuroinflammation, pain, and oxidative stress. It also decreased the expression of ROS and pro-inflammatory cytokines [37], in addition to its protective and antinociceptive effects [38].

Considering the evidence collected about these natural extracts and their properties related to the specific intracellular mechanisms involved in NP, this study attempts to demonstrate the positive effects of the formulation consisting of PEA, Ginkgo Biloba, Hops, Propolis, and Vitamin B in vitro model starting from an intestinal absorption to reach peripheral nerve 3D model after damages mimicking the NP condition. This study aimed particularly to demonstrate for the first time the beneficial effects that could be exerted by a single agent and as the innovative combination to enhance the beneficial effects of the single one, analysing their possible synergistic effect.

## 2. Results

### 2.1. Dose-Response Assessment Study of Single Components on CaCo-2 Cells

**Figure 1:**
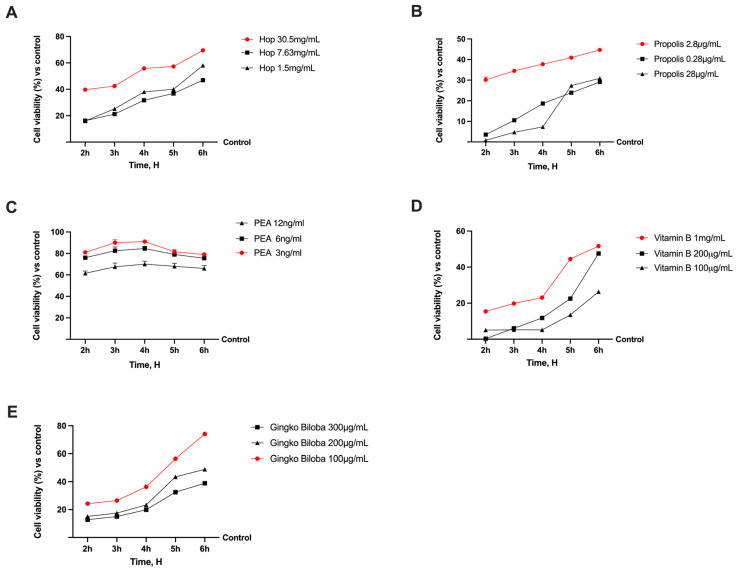
Analysis of different substances in a dose-response and time-dependent study evaluating cell viability by MTT test. In (**A**) Hop Extract; (**B**) Vitamin B group; (**C**) PEA 80 mesh; (**D**) Propolis 4.5% Artepillin; (**E**) Gingko Biloba effects on cell viability. Results are normalised to control (untreated sample) value (0%) to explain percentage increase or decrease in effects of substances tested. Data expressed as mean ± SD (%) of five independent experiments normalised to control (0%) line. All concentrations in red are *p* < 0.05 vs. control.

Before exploring the beneficial effects of the new formulation in the context of NP and, specifically, nerve degeneration, the study’s first phase consisted of evaluating and characterising the effects of the natural components of interest on CaCo-2 cells. Specifically, a dose-response study highlighted the best concentration for each substance tested, considering a period between 2 and 6 h as the relevant exposure time. As can be seen from Figure 1, every substance increased CaCo-2 cell viability in comparison to the control (*p* < 0.05). Specifically, Hops extract was tested at different concentrations ranging from 1.5 to 30.5 mg/mL, and the higher concentrations revealed a better profile in terms of cell viability compared to the other lower concentrations tested. In addition, a similar trend was observed with Vitamin B, where Propolis showed that in this case, the higher concentration exerted had the most beneficial effects. On the other hand, the lowest concentrations tested of PEA and Gingko Biloba demonstrated a better trend than the other concentrations chosen. Thus, based on the results obtained, the hypothesized formulation is Hop 30.5 mg/mL, Propolis 2.8 µg/mL, Ginkgo Biloba 100 µg/mL, Vitamin B 1 mg/mL, and PEA 3 ng/mL.

### 2.2. Functional Assessment at Intestinal Level

**Figure 2:**
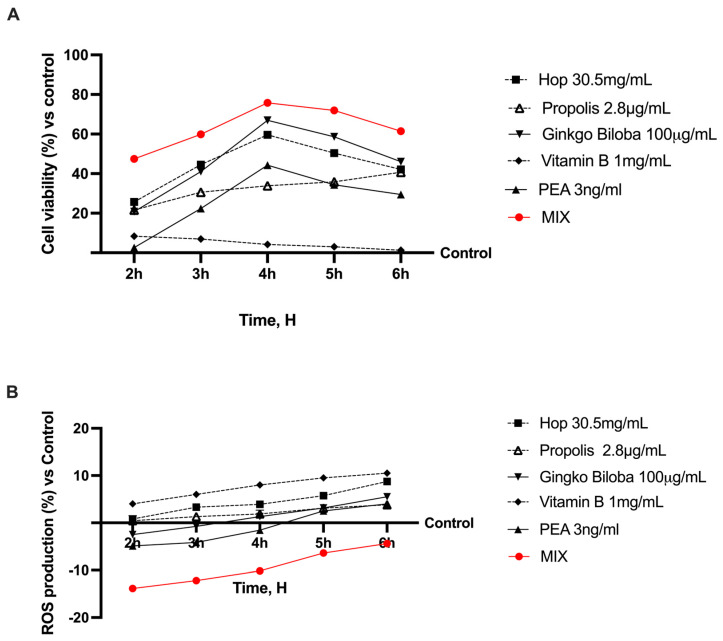
Analysis of cell viability (**A**) and ROS production (**B**) of single components and their combinations in time-dependent study. MIX = Hop 30.5 mg/mL + Propolis 2.8 µg/mL + Ginkgo Biloba 100 µg/mL + Vitamin B 1 mg/mL + PEA 3 ng/mL. Results are normalised to control (untreated sample) value (0%) to explain percentage increase or decrease in effects of substances tested. Data are expressed as mean ± SD (%) of five independent experiments normalised to control. All concentrations in red are *p* < 0.05 vs. control except 6 h of ROS production analysis.

Subsequently, several experiments were performed on a 3D in vitro model of the intestinal barrier to exclude any cytotoxic effect and confirm the absence of oxidative stress by comparing the concentrations of the selected individual natural components with their combination. As shown in Figure 2, all the selected agents maintained a balanced gut environment with good levels of cell viability and oxidative stress production. Compared with the single agents and untreated cells (control), statistically better values were obtained after stimulation with the combination of Hop 30.5 mg/mL, Propolis 2.8 µg/mL, Ginkgo Biloba 100 µg/mL, Vitamin B 1 mg/mL, and PEA 3 ng/mL (named as MIX). Indeed, MIX did not cause any harm at the intestinal level but enhanced the beneficial effects observed with the individual compounds (*p* < 0.05). In particular, MIX was able to improve cell viability without toxic effects and without inducing oxidative stress. Therefore, based on these results, it can be considered that MIX and all selected agents can positively affect the intestinal barrier without negative side effects after its intake.

**Figure 3:**
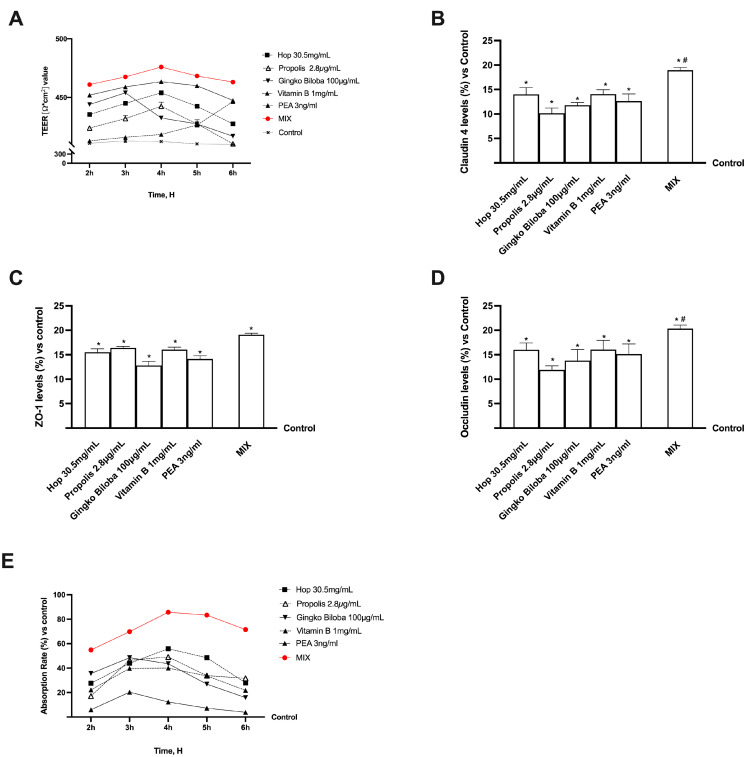
Permeability study on CaCo-2 cells. In (**A**) TEER (transepithelial electrical resistance) value using EVOM3; from (**B**–**D**) analysis of TJ measured by enzyme-linked immunosorbent assay (ELISA) test (Occludin, Claudin1, and ZO-1 respectively); in (**E**) absorption rate represented by Papp normalised to control (%). Abbreviations correspond to those used in Figure 2. From (**B**–**D**), means ± SD are expressed comparing data to control (0% line), * *p* < 0.05 vs. control; # *p* < 0.05 vs. single agents. In (**A**–**E**) # *p* < 0.05 vs. single agents.

Further research used a 3D in vitro intestinal barrier model to obtain significant insights into intestinal absorption and transport mechanisms. Specifically, the transepithelial electrical resistance (TEER), intestinal absorption, and TJ proteins, including Occludin, Claudin-1, and Zonula occludens-1 (ZO-1), were evaluated. As shown in Figure 3A, the substances tested, alone or in combination, support the correct homeostasis of intestinal CaCo-2 cells, as can be deduced by the TEER values higher than the control and established cut-off point for appropriate intestinal barrier integrity. Furthermore, as inferred from the previously collected data, MIX could preserve epithelial integrity and raise the ion flux of paracellular exchanges across the intestinal epithelium while improving the absorption rate with physiological behaviour, even regarding individual components (*p* < 0.05). The analysis of tight junction (TJ) proteins such as Occludin further confirmed these data, which aid in stabilization, adhesion-mediating ZO-1, and structure-maintaining Claudin (Figure 3B–D). Even in this case, combining the agents better ameliorates the TJs function than their single action (*p* < 0.05).

Additional investigations were conducted to obtain further information on intestinal absorption by analysing apparent permeability (Papp) coefficient values to measure the absorption rate and predict bioavailability. Therefore, the flow of nonelectrolyte tracers (expressed as the Papp coefficient) that crossed the intestinal barrier was analysed by fluorescence analysis. The data shown in Figure 3E confirm the intestinal absorption. In particular, the rate of MIX was greater than each compound with a peak absorption around 4 h (*p* < 0.05) and maintained this effect by decreasing slowly over time (*p* < 0.05) suggesting a synergic effect of the agents.

### 2.3. Effects of Single Components and Combination on 3D EngNT Cocultures

**Figure 4:**
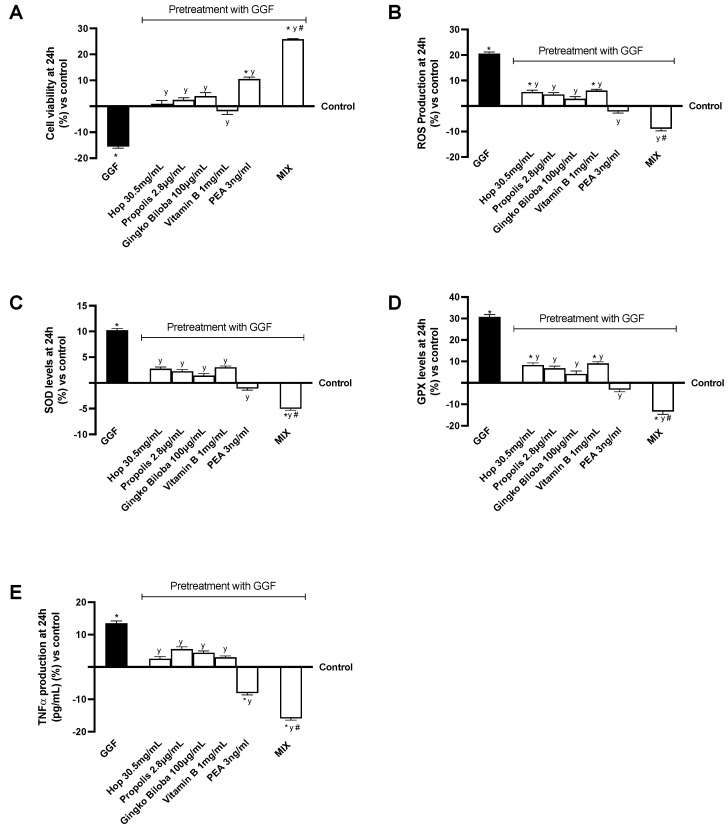
Analysis of 3D EngNT under PNI conditions. (**A**) Cell viability measured by MTT test; (**B**) analysis of ROS production measured by cytochrome C reduction; (**C**) SOD levels; (**D**) GPX levels; (**E**) TNFα quantification by ELISA kit. Data are the mean ± SD of five independent experiments performed in triplicate and normalised to control values (0% line) to explain results as a percentage of increase or decrease. MIX = Hop extract 30.5 mg/mL + Propolis 4.5% Artepillin 2.8 µg/mL + Ginkgo Biloba 100 µg/mL + Vitamin B 1 mg/mL + PEA 3 ng/mL; GGF = glial growth factor 2. * *p* < 0.05 vs. control; # *p* < 0.05 vs. single agents; y *p* < 0.05 vs. GGF.

To simulate peripheral nerve tissue injury in vitro, EngNT 3D was pretreated with 200 ng/mL glial growth factor 2 (GGF) beginning on day 14 of maturation to induce robust demyelination before stimulation with the same agents previously employed. Additional tests were carried out to examine the impact on mitochondrial metabolism and the release of ROS in this scenario as reported in Figure 4A,B. Nerve tissue treated with 200 ng/mL GGF showed a substantial decrease in nerve biological activity and increased production of ROS compared to the control group (*p* < 0.05). On the other hand, the negative conditions were effectively neutralised by all drugs examined individually (*p* < 0.0001). In particular, MIX statistically significantly improved cell viability compared to the individual substances (*p* < 0.0001). At the same time, it was also able to reduce oxidative stress (*p* < 0.0001) produced during damage conditions, supporting the important results previously observed on the synergistic effect of the substances. To complete the picture on the analysis of the substances tested on the potential antioxidant activity, the levels of SOD, a very important antioxidant defence against oxidative stress in the body, and GPX, a family of enzymes that protects cells from oxidative stress, were carried out (Figure 4C,D). Both analyses confirmed the antioxidant activity, strongly reducing the levels of oxidative stress induced by GGF pre-treatment (*p* < 0.0001). Among the individual agents, for both SOD and GPX levels, PEA exerts the greatest effect by completely reversing the effects caused by GGF (*p* < 0.001). As expected, the action of the mix turned out to be even more effective as it decreased SOD and GPX levels even significantly compared to the control as well as individual agents and GGF (*p* < 0.0001). Likewise, it can be observed that GGF produced a major inflammatory state mediated by high tumor necrosis factor α (TNFα) production (*p* < 0.05) compared with the untreated control (Figure 4E). Even in this case, this effect was counteracted by the individual substances and MIX (*p* < 0.0001), thus supporting the slowing in damage progression by exploiting physiological recovery mechanisms.

**Figure 5:**
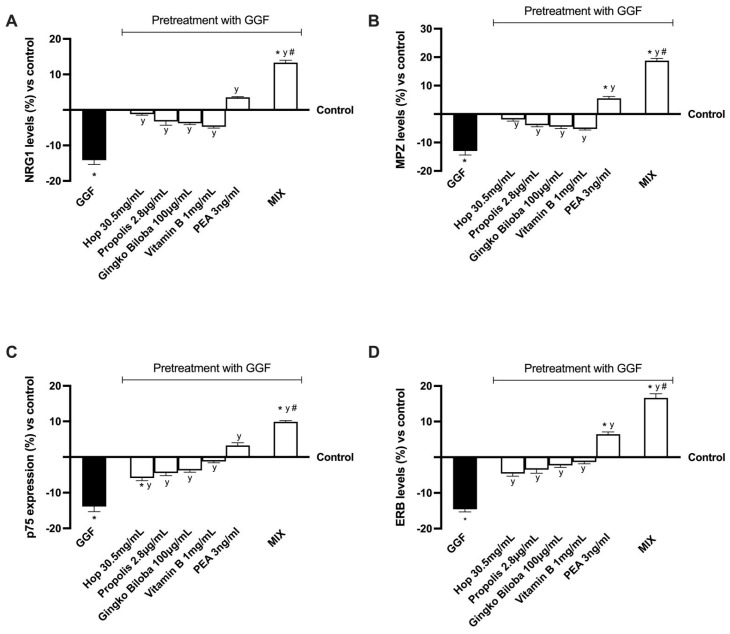
Analysis of molecular markers on 3D EngNT under PNI conditions. (**A**) NRG1, (**B**) MPZ, (**C**) p75, and (**D**) ERb were measured by ELISA test. Data are means ± SD (%) of five independent experiments performed in triplicate and normalised to control values (0% line) to explain the results as percentage of increase or decrease. Abbreviations correspond to those used in Figure 4. * *p* < 0.05 vs. control; # *p* < 0.05 vs. single agents; y *p* < 0.05 vs. GGF.

Finally, the regulatory mechanisms underlying the regulation of specific molecular pathways related to NP were analysed following treatment with 200 ng/mL GGF (Figure 5). As can be observed in Figure 5A, following the treatment with the tested agents, alone or in combination, there is an amelioration of nerve injury. Further, following the treatment with MIX, a significant effect is evident compared to the non-treated cells (about 15% compared to control, *p* < 0.0001), also than compared to all the single agents (*p* < 0.0001). This pattern was observed also analysing myelin protein zero (MPZ) level, a protein involved in maintaining the myelin sheath. All the agents improved the MPZ levels compared to GGF (*p* < 0.001), indicating the restoration of the myelin sheath. Moreover, MIX induced the greatest effects compared with the individual substances tested (*p* < 0.0001), reversing the induced damage. Also, the analysis of p75 expression confirmed these data. After the pre-treatment with GGF, myelinating cells were degraded, but this condition was restored following treatment with the tested agents, alone and in combination (*p* < 0.001). Specifically, they maintained the myelin sheath at normal activity, enhancing the expression of p75 (*p* < 0.001). Notably, when compared to the individual agents examined, MIX had the greatest effects (*p* < 0.001), successfully correcting the damage and inducing an activity restoration even compared to the control (about 11%, *p* < 0.0001). Finally, the treatment with all the agents tested impacted the epidermal receptor beta (ERb) level. All examined substances individually reduced damage induced by GGF, indicating their beneficial role in preventing demyelination (*p* < 0.001). At the same time, the level of ERb increased in the presence of MIX compared with single substances, supporting the hypothesis of enhancing this marker to improve nerve injury and restore the myelination process. In addition, MIX’s active involvement is verified by the appropriate synergistic effect that enabled it to influence several components of damage progression, ultimately slowing down the process and reducing the condition that is involved in neuropathy development.

## 3. Discussion

Managing NP is a prominent obstacle in contemporary medicine. Traditional medicine has utilised natural chemicals such as nutraceuticals to address this issue. Substantial evidence has demonstrated their effectiveness in managing oxidative stress and chronic pain-related inflammation [39]. Based on the numerous important findings reported in the literature about the antioxidant and antinociceptive properties of the substances selected, this study permits the preliminary exploration of the main target involved in NP. In particular, PEA revealed much stronger antioxidant activity than the other agents tested alone, with appropriate anti-inflammatory potency based on TNFα marker study. In contrast, Hop extract only had an anti-inflammatory impact, although Gingko Biloba, Propolis, and Vitamin B had very similar antioxidant and anti-inflammatory effects, albeit at a lesser level than PEA. The novel formulation performed better in maintaining adequate intestinal barrier function and stability while exhibiting no deleterious effects. Indeed, the data gathered revealed an additive impact through MIX. At the peripheral nerve level, examination of markers such as NRG1 revealed that MPZ had stronger supporting action by PEA due to enhanced activation of pathways that promote neuropathic regeneration. Hop extract appears to increase NRG1 expression while decreasing demyelination at the nerve level, but does not improve p75 and Erb levels, which are directly connected to healthy peripheral nerve function. Propolis, Gingko Biloba, and B Vitamins showed no significant changes at the peripheral nerve level in this setting. The new formulation significantly preserved and sustained adequate peripheral nerve function and stability. In particular, the findings obtained revealed a synergistic effect due to MIX. In addition, the topic of application is relevant, since this NP is common in a population of middle- to older-aged people with mixed aetiologies [5]. Nutraceutical could be clinically relevant for the management of the disease [39].

NP can be caused by diseases or lesions of the somatosensory nervous system, either centrally or peripherally [40]. NP is clinically defined by induced heightened pain responses and spontaneous continuous or shooting pain when exposed to either toxic or non-noxious stimuli [9]. Due to basic research, different pathophysiological mechanisms are becoming increasingly identifiable, and the clinical evaluation of symptoms and indications can aid in identifying the mechanisms responsible for various neuropathy syndromes [41]. Peripheral neuropathies have been identified as the clinical manifestation of dysfunction in the Schwann cells that encase peripheral axons and/or axons themselves [12]. Furthermore, there is a developing link between peripheral neuropathies and oxidative stress. It is widely accepted that oxidative stress occurs when the ratio of beneficial antioxidants to damaging free radicals is imbalanced and tilts toward oxidation. ROS are the most common type of free radical, and have the potential to initiate molecular instability chain events. Lipid peroxidation is an example of this, in which the cell’s lipid bilayer membranes undergo this chain of events [13]. The largest source of ROS in the body is aerobic respiration. However, ROS is also produced by peroxisomal-b oxidation of fatty acids, pathogen or lipopolysaccharide-stimulated phagocytosis, arginine metabolism, and tissue-specific enzymes. ROS, when unregulated, destroys various essential components, including enzymes, membranes, lipids, and DNA. SOD, catalase, or glutathione, as well as antioxidant Vitamins C and E, remove ROS from cells under normal conditions [14]. Several animal models have been established to investigate the processes of peripheral neuropathy over time. The oxidative stress theory appears to be gathering progress in this context. For instance, the idea that neuropathies like tinnitus are characterised by elevated oxidative stress was developed after a study connected this kind of neuropathy to greater levels of free radicals and plasma scavenger activity [15]. This explains why several natural antioxidant therapies are gaining traction as cutting-edge approaches to treating NP. Studies have shown that antioxidants can have preventive and protective effects by reducing the body’s generation of free radicals, which might be damaging neurons [18]. Thus, this study aimed to examine the impact of specific antioxidant substances on the discomfort associated with chronic NP and the relationship with peripheral nerve damage. Indeed, the combined use of plant extracts may improve the efficacy by achieving synergy, operating on several targets at the same time, lowering individual component doses, and reducing adverse effects [42].

Translating our assumptions into practice, we investigated the novel formulation’s biological mode of action using an in vitro experimental model miming intestinal environment. Our findings demonstrated that the novel nutraceutical could regulate the primary processes responsible for nerve fibre deterioration, which is responsible for some neuropathic diseases. Specifically, the integration with a combination of Hop 30.5 mg/mL, Propolis 2.8 µg/mL, Ginkgo Biloba 100 µg/mL, Vitamin B 1 mg/mL, and PEA 3 ng/mL was able to reduce the oxidative stress produced after the induced damage. This capacity is probably mainly due to the high proportion of polyphenols contained within this MIX, as it has been proven that polyphenols are efficient antioxidants in various chemical oxidation systems due to their capacity to scavenge ROS [43]. Indeed, dietary polyphenols include several hydroxyl groups, an aromatic structural characteristic, and a highly conjugated system that enables them to function as effective free radicals and ROS scavengers, as per the biochemical scavenger theory. Their ability to neutralise ROS or inhibit cellular oxidative stress prevents oxidative damage to biomolecules, reducing tissue inflammation [44]. All substances tested (apart from B vitamins) contain many flavonoids; therefore, their activity in reducing ROS production was highly expected. In addition, the potential antioxidant properties of B12 are involved in the direct removal of ROS, so again the single agent directly affects oxidative stress.

These effects were greatly enhanced by MIX, thus demonstrating the direct effect on oxidative stress levels. This antioxidant picture was also confirmed by SOD and GPX analysis, demonstrating the strong antioxidant capacity of MIX. These data are of major relevance because SOD and GPX can directly counteract the attack of oxidants and protect cells from DNA damage [45]. Polyphenols have also been shown to have anti-inflammatory effects through this scavenger mechanism and the modulation of the production of other proinflammatory molecules [46]. In this context, the anti-inflammatory effect is essential for modulating NP. Indeed, studies have shown that an elevated level of TNFα and its receptor is found at sites of nerve damage in animal models of NP [47,48]. In addition, it was found that the administration of TNFα antagonists decreased behaviours suggestive of pain [49]. Indeed, in this study, TNFα production was also found to be modulated after stimulation with the MIX examined, indicating the possible functionality of this treatment in the context of NP by confirming the anti-inflammatory action of the selected substances like Gingko Biloba [50], PEA [51], and Hop extract [52] described in the literature in numerous studies in this regard. Therefore, the following experiments aimed at understanding whether the combination of Hop 30.5 mg/mL, Propolis 2.8 µg/mL, Ginkgo Biloba 100 µg/mL, Vitamin B 1 mg/mL, and PEA 3 ng/mL is effective in treating NP were carried out on a peripheral nerve model under damaged conditions. The data gathered demonstrated that the MIX can repair damage to the myelin sheath that protects the axon. Indeed, MIX restored NRG1 level, a protein that plays a key role in the early stage of the injury at the peripheral nerve and whose lack of Schwann cells strongly impaired nerve remyelination. NRG1 level is related to ERb receptors as normal peripheral nerve growth, migration, differentiation, and dedifferentiation of Schwann cells are regulated by NRG1 activating ERb receptors [53]. On the other hand, this signaling is modified during nerve damage through the imbalance between NRG1 isoforms [54]. While the disruption of NRG1/ERb signaling and impaired neurotrophic support are undoubtedly linked to the degeneration of Schwann cells, treatment with the MIX of Hop 30.5 mg/mL, Propolis 2.8 µg/mL, Ginkgo Biloba 100 µg/mL, Vitamin B 1 mg/mL, and PEA 3 ng/mL restored the altered neurotropism. Additionally, p75 expression measurement demonstrated the effectiveness of this treatment as axonal degeneration and dysfunction during injury and cellular stress are caused by certain signaling molecules, including p75, whose expression rises dramatically after injury [55]. Finally, further evidence of the effectiveness of the combination of Hop 30.5 mg/mL, Propolis 2.8 µg/mL, Ginkgo Biloba 100 µg/mL, Vitamin B 1 mg/mL, and PEA 3 ng/mL is provided by the analysis of MPZ, a component of the myelin that undergoes at upregulation at the start of the myelination process forming a membrane structure and whose expression is decreased after peripheral nerve injury [56].

These results endorse the potential application of a novel combination designed for crossing biological membranes, specifically the intestinal barrier, to reach the target area. Indeed, the effects of Hop 30.5 mg/mL, Propolis 2.8 µg/mL, Ginkgo Biloba 100 µg/mL, Vitamin B 1 mg/mL, and PEA 3 ng/mL combination were analyzed by mimicking human oral administration in vitro. The findings from experiments using the 3D model showed the originality of this unique formulation, which allows effective delivery to the peripheral nervous system, where the nerve damage typical of peripheral neuropathy is present. The two in vitro models used are selected for their specific characteristics. In particular, Caco-2 cell line permits examining the intestinal permeability using a standard method approved by EMA and FDA, and the RSC-96 and PC12 cells are well known to study peripheral neuropathy as reported in the literature. However, considering that it is a preliminary study conducted in vitro, more in-depth studies are needed to establish the effectiveness of this new combination, for example, integrating the model used with the dorsal root ganglion to explore the sensory electrical stimulation. Nevertheless, it is essential to acknowledge that NP is a complex phenomenon and that existing models must be refined further to enhance their predictive power.

## 4. Materials and Methods

### 4.1. Agents Preparation

To find out if a new formulation could slow down nerve injury or damage in the peripheral nervous system, the following substances were tested: 3 ng/mL PEA, Hop extract 30.5 mg/mL, Propolis 4.5% Artepillin 2.8 µg/mL, Ginkgo Biloba 100 µg/mL, Vitamin B (mix of 22.5% of Vitamin B1 and B2, 16.2% of Vitamin B5, 9% of Vitamin B6 and 29.7% of Vitamin B12) 1 mg/mL, both individually and in combination. The tested concentrations of PEA, Hop, Propolis, Ginkgo Biloba, and Vitamin B were determined from the literature [57,58] and validated through dose-response studies. These studies employed concentrations of PEA ranging from 3 ng/mL to 12 ng/mL, Hop from 1.5 mg/mL to 30.5 mg/mL, Propolis from 0.28 µg/mL to 28 µg/mL, Ginkgo Biloba from 100 µg/mL to 300 µg/mL, and Vitamin B from 100 µg/mL to 1 mg/mL. Vivatis Pharma Italia S.r.l. (Gallarate, VA, Italy) provided the tested substances as donations and they were prepared in Dulbecco’s Modified Eagle’s Medium (DMEM provided by Merck Life Science in Rome, Italy) supplemented with 0.5% foetal bovine serum (FBS), 2 mM L-glutamine, and 1% penicillin–streptomycin (all component of the preparation medium were from Merck Life Science in Rome, Italy). 200 ng/mL GGF (Tebu-Bio, Magenta, Milan, Italy) was added directly to the medium into the 3D EngNT to induce demyelination. When the better concentration was selected for each agent, their combination was tested as treatment in the context of peripheral damage.

### 4.2. Cell Culture

The CaCo-2 cell line from the American Type Culture Collection (ATCC) was utilised as an experimental model to predict the characteristics of substances absorbed by the intestinal tract [59,60]. This cell line was grown in Dulbecco’s Modified Eagle’s Medium Advance (DMEM-Adv, Thermo Fisher Scientific, Rodano, MI, Italy), which contained 1% penicillin-streptomycin (Merck Life Science, Rome, Italy), 5% FBS (Merck Life Science, Rome, Italy), and 2 mM L-glutamine. The culture was kept in an incubator at 37 °C and 5% CO_2_ [61]. For proper paracellular permeability and transport, experiments used cells between 26 and 32 at passage numbers [62]. Depending on the test performed, the cells were plated differently: 1 × 10^4^ cells on 96-well plates were used to study cell viability and ROS production. The cells were synchronised with DMEM without red phenol for 8 h and then supplemented with 0.5% FBS, 2 mM L-glutamine, and 1% penicillin–streptomycin at 37 °C. An amount of 2 × 10^4^ cells on 6.5 mm Transwell^®^ (Corning^®^ Costar^®^, Merck Life Science, Rome, Italy) with a 0.4 μm pore polycarbonate membrane insert were used for integrity and absorption studies [63].

The RSC-96 cell line derived from rats, purchased from ATCC, was cultured in DMEM-Adv (Merck Life Science, Rome, Italy) containing 5% FBS (Merck Life Science, Rome, Italy), 2 mM L-glutamine, and 1% penicillin–streptomycin (Merck Life Science, Rome, Italy) [64]. The cultures were maintained at 37 °C with 5% CO_2_. Experiments utilised RSC96 cells that had been sub-cultured 2 to 3 times per week in passages ranging from 10 to 15 [65].

Rat neuronal PC12 cells (from ATCC), commonly used for in vitro screening of neuroprotective compounds [66], were cultured in Roswell Park Memorial Institute-1640 Advance (RPMI-Adv, provided by Merck Life Science in Rome, Italy) with 5% horse serum (HS,Merck Life Science, Rome, Italy), 2.5% FBS, and 2 mM glutamine (Merck Life Science, Rome, Italy). The cell lines utilised in the studies were from passages 3–13, and the cultures were kept at sub-confluency at 37 °C and 5% CO_2_ [67]. An amount of 4 × 10^6^ RSC96 cells and 1 × 10^5^ PC12 cells were co-cultured to reproduce 3D EngNT in vitro in the peripheral nerve environment [68].

### 4.3. Experimental Procedure

**Figure 6:**
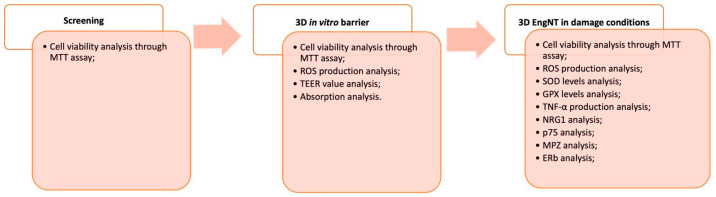
Schematic diagram of experimental protocol subdivided into phases with specific method used.

The study was subdivided into three steps (Figure 6). The first examined the minimal effective dosage of PEA (3 ng/mL; 6 ng/mL; 12 ng/mL), Hop (30.5 mg/mL; 7.63 mg/mL; 1.5 mg/mL), Propolis (28 µg/mL; 2.8 µg/mL; 0.28 µg/mL), Ginkgo Biloba (300 µg/mL; 200 µg/mL; 100 µg/mL), and Vitamin B (1 mg/mL; 200 µg/mL 100 µg/mL) on CaCo-2 cells in a dose-response study. Subsequently, better concentrations of all substances alone and combined were used in a 3D in vitro model to test the formulation and individual components across the intestinal barrier, analysing safety and toxicity, oxidative stress, barrier integrity, TJs proteins, and absorption prediction [68]. Specifically, cells were plated in the Transwell^®^ system after the treatments to confirm intestinal integrity via TEER assessment. Furthermore, the absorption rate was assessed using the Papp analysis, and TJ levels were examined using ELISA in this in vitro intestinal model. These tests were time-dependent, ranging from 2 to 6 h [68]. In addition, the basolateral environment was gathered for use in the 3D EngNT co-culture after every simulation. The effects of the stimulations on the nerve tissue model were examined in vitro using a 3D EngNT co-culture in the third and final stage, which followed a 24 h treatment period and a 14 d culture maturation period. This method was developed to simulate peripheral nerve injury and demyelination using GGF 200 ng/mL [69]. Cell viability, oxidative by analysing ROS, SOD, and GPX, and inflammatory status through the TNFα test, and the primary pathways in myelin sheath protection and neurite formation processes, such as NRG1, MPZ, p75, and ERb, were examined in this phase after the induction with GGF 200 ng/mL.

### 4.4. In Vitro Simulation of Intestinal Barrier

An in vitro intestinal barrier model was established using the Transwell^®^ system to assess the ability of PEA, Hop, Propolis, Ginkgo Biloba, and Vitamin B samples to pass through the intestinal barrier. The protocol used following guidelines [70,71] from the European Medicines Agency (EMA) and the Food and Drug Administration (FDA) for predicting the absorption, metabolism, and bioavailability of substances following oral ingestion in humans [72,73]. Before simulations, CaCo-2 cells, plated as described, were maintained in a full medium and changed every other day on the basolateral and apical sides for 21 days [70]. To analyse the development of mature intestinal epithelial cells and an appropriate paracellular mechanism, the TEER values were measured throughout the entire maturation period using EVOM3 in conjunction with STX2 chopstick electrodes (World Precision Instruments, Sarasota, FL, USA). Absorption analysis began on day 21 when TEER values were ≥400 Ω cm^2^ [74]. Before the stimulation, the medium was adjusted to the pH of the small intestine lumen (pH 6.5) on the apical side and blood (pH 7.3) on the basolateral side [62,75]. The cells were stimulated with all substances plus 0.04% fluorescein (Santa Cruz, CA, USA) from 2 to 6 h before the successive analyses, including the absorption rate by Papp analysis following the formula:

Papp = [V_a_/(Area × time)] × ([drug]_acceptor_/[drug]_initial,donor_)

V_a_: volume in the acceptor well (in mL);

Area: surface area of the membrane;

time: total transport time in seconds.

Negative controls without cells were tested to rule out Transwell^®^ membrane effects. Analysis was carried out in triplicates and repeated five times.

### 4.5. 3D EngNT Co-Cultures Setup

The 3D nerve tissue model was developed based on the literature [64]. Interactions between RSC96 and PC12 cell lines are crucial for replicating the peripheral nerve environment in vitro, promoting neurite regeneration, and supporting Schwann cells [64,76,77]. In summary, a rectangular scaffold measuring 16.4 mm × 6.5 mm × 5 mm was filled with 1 mL of a solution that contained 80% *v*/*v* Type I rat tail collagen (2 mg/mL in 0.6% acetic acid, Thermo Fischer, Milan, Italy), 10% *v*/*v* Minimum Essential Medium (MEM, Merck Life Science, Milano, Italy), 5.8% *v*/*v* neutralizing solution (Biosystems, Monza, Italy), and 4.2% Schwann cell suspension (4 × 10^6^ RSC96 cells per 1 mL gel). After the gel solidified, it was submerged in 10 mL of Dulbecco’s Modified Eagle’s Medium (DMEM, Merck Life Science, Rome, Italy) with red phenol and supplemented with 10% FBS, 100 U/mL of Penicillin, and 100 μg/mL of Streptomycin (Merck Life Science, Milano, Italy) for 24 h at 37 °C with 5% CO_2_. Upon completion of the incubation period, the gel was stabilised using plastic compression (120 g of weight for one minute). The gel was divided into equal parts based on the samples to be treated once it had been aligned and stabilised. After the aligned Schwann gels, each gel segment was moved to a 24-well plate. To construct the co-cultures, 1 × 10^5^ PC12 was seeded on top of each segment. This passage is crucial since it enables neurite outgrowth horizontally. After allowing neuronal cells to attach to the collagen gel by incubating the 24-well plate with gels for one hour at 37 °C, 1 mL of culture medium was added to each well.

### 4.6. Cell Viability

After each stimulation, the cell viability was assessed using the MTT In Vitro Toxicology Assay Kit (Merck Life Science, Rome, Italy), following a traditional technique [70]. The absorbance of all solubilised samples—treated and untreated—was measured at 570 nm with correction at 690 nm using a spectrometer (Infinite 200 Pro MPlex, Tecan, Männedorf, Switzerland). Five independent tests were conducted in triplicate, and the means of these experiments were reported. The data was expressed by comparing the results to the control sample, which was an untreated sample defined as the 0% line.

### 4.7. ROS Production

By measuring the absorbance at 550 nm with a spectrometer (Infinite 200 Pro MPlex, Tecan, Mannedorf, Switzerland), the ROS production was quantified via analysis of the reduction of cytochrome C following a standard protocol [61]. In five separate studies conducted in triplicate, the O_2_ ratio was expressed as the mean ± SD (%) of nanomoles per decreased cytochrome C per microgram of protein relative to the control (untreated samples).

### 4.8. TJs Analysis

The human occludin (OCLN) ELISA kit (MyBiosource, San Diego, CA, USA), claudin-1 (ELISA kit, Cusabio Technology LCC, Houston, TX, USA), and ZO-1 (human tight junction protein 1 (TJP1) ELISA kit (MyBiosource, San Diego, CA, USA) were used to analyse the CaCo-2 lysates following the manufacturer instructions. The spectrophotometer used to measure absorbance was the Infinite 200 Pro MPlex from Tecan, located in Männedorf, Switzerland, operating at a wavelength of 450 nm. The data were acquired by comparing the standard curve ranging from 0 to 1500 pg/mL for occludin, and from 0 to 1000 pg/mL for claudin-1 and ZO-1. The data was shown as a percentage compared to the control (0 line) from five independent experiments conducted in triplicates [61].

### 4.9. SOD Assay

The level of SOD was measured following the manufacturer’s instructions (Cayman’s Superoxide Dismutase Assay Kit; Tallinn, Estonia) on 3D EngNT lysate [63]. The level of SOD present on 3D cell lysates was measured by comparing data to a standard curve (0.05–0.005 U/mL). The absorbance of all samples was measured through a spectrometer (Infinite 200 Pro MPlex, Tecan, Männedorf, Switzerland) at 440–460 nm, and results were expressed as means (%) compared to the control.

### 4.10. Glutathione Peroxidase Assay

The level of GPX was measured on 3D EngNT lysate following the manufacturer’s instructions (Cayman’s Superoxide Dismutase Assay Kit; Tallinn, Estonia). Briefly, in the positive control well were added 50 µL of Assay Buffer, 50 µL of Co-Substrate Mixture, 50 µL NADPH, and 20 µL of diluted GPX properly diluted; in the sample wells were added 50 µL of Assay Buffer, 50 µL of Co-Substrate Mixture, 50 µL NADPH, and 20 µL of the tested sample. After adding 20 µL of Cumene Hydroperoxide to the wells, the plate was gently shaken for a few seconds. Finally, the level of GPX present on 3D cell lysates was measured by comparing the data to the positive control. The absorbance of all samples was measured through a spectrometer (Infinite 200 Pro MPlex, Tecan, Männedorf, Switzerland) at 340 nm and the results were expressed as means (%) compared to the control.

### 4.11. TNFα Assay

Following the manufacturer’s instructions, TNFα quantification was achieved using the TNFα ELISA kit (Merck Life Science, Milano, Italy) on 3D EngNT culture supernatants [78]. Sample absorbance was measured at 450 nm using a plate reader (Infinite 200 Pro MPlex, Tecan, Männedorf, Switzerland). Five independent experiments were conducted in triplicate, and the findings were expressed as a mean SD (%) vs. the control (0 line).

### 4.12. NRG1 Assay

The NRG1 Rat ELISA Kit (FineTest, Wuhan, China) was utilised in 3D EngNT cell culture supernatants following the manufacturer’s instructions [79]. A plate reader (Infinite 200 Pro MPlex, Tecan, Männedorf, Switzerland) measured sample absorbance at 450 nm. The collected data were compared to the standard curve, which ranged from 0.156 to 10 ng/mL. The results were expressed as mean SD (%) of five independent tests conducted in triplicate against the control (0 line).

### 4.13. Myelin Protein Zero Assay

As directed by the manufacturer, the MPZ level in 3D EngNT cell lysates was assessed using a Rat ELISA kit (MyBiosource, San Diego, CA, USA) [79]. The samples were read at 450 nm by a spectrometer (Infinite 200 Pro MPlex, Tecan, Männedorf, Switzerland), and results were presented as the mean SD (%) vs. the control (0 line) of five independent tests carried out in triplicates. The concentration was indicated as ng/mL with a standard curve (range from 0.06 to 18 ng/mL).

### 4.14. NGFR Assay (p75 Expression Assay)

Based on product instructions, the Rat NGFR ELISA kit (MyBiosource, San Diego, CA, USA) was utilised on 3D EngNT cell lysates [79], reading the samples at 450 nm in a spectrometer (Infinite 200 Pro MPlex, Tecan). The data were compared to the standard curve (0.312–20 ng/mL) and presented as a mean SD (%) versus control (0 line) from five independent experiments in triplicates.

### 4.15. Estrogen Receptor Beta Assay

According to the manufacturer, 3D EngNT cell lysates were tested with the Rat Oestrogen Receptor Beta (ERb) ELISA Kit (Cloud-Clone, Houston, TX, USA) [80]. In summary, 100 µL of each sample was put in each well and incubated at 37 °C for 1 h. After all reactions, 50 µL of Stop Solution was added to each well, and the plate was scanned at 450 nm using a spectrometer (Infinite 200 Pro MPlex, Tecan). The concentration was compared to a standard curve (0.312–20 ng/mL) and showed as mean ± SD (%) versus control in five independent experiments conducted in triplicates.

### 4.16. Statistical Analysis

A minimum of five separate triplicate experiments provided the data for this investigation. Data were examined using Prism GraphPad. Results are reported as means ± standard deviation (%) via One-way Analysis of Variance (ANOVA) and Bonferroni post hoc test for statistical analysis. The significance level was set at *p* < 0.05.

## 5. Conclusions

NP is commonly caused by damage to the peripheral nerves, a condition usually dependent on peripheral neuropathy related to oxidative stress and inflammation. Thus, this study explores the combined ability of Hop, Propolis, Ginkgo Biloba, Vitamin B, and PEA to reduce oxidative stress and inflammation produced after nerve damage, as well as to modulate the key mechanisms involved in the recovery such as NRG1, MPZ, ERB, and p75 levels. This treatment can potentially restore the myelin sheath and, therefore, could be considered an innovative strategy for NP management. Specifically, it is worth noting that MIX has a more promising effect on neuropathic pain than every single agent tested alone.

## Data Availability

The Laboratory of Physiology (C. Molinari) collects raw data and takes appropriate procedures to preserve them in a secure system forever. The corresponding author can provide this study’s data upon reasonable request.

## Abbreviations

CCI	chronic nerve constriction injury
DMEM	Dulbecco’s Modified Eagle’s Medium
DMEM-Adv	Dulbecco’s Modified Eagle’s Medium Advance
ERb	epidermal receptor beta
EMA	European Medicines Agency
FBS	foetal bovine serum
FDA	Food and Drug Administration
GGF	glial growth factor 2
HS	horse serum
MEM	Minimum Essential Medium
MPZ	myelin protein zero
NP	neuropathic pain
Nrf2	nuclear factor erythroid 2-related factor 2
Papp	apparent permeability coefficient
PCPA	P-chlorophenylalanine
PEA	palmitoylethanolamide
ROS	reactive oxygen species
RPMI-Adv	Roswell Park Memorial Institute-1640 Advance
SOD	superoxide dismutase
TJ	tight junction
TEER	transepithelial electrical resistance
TNFα	tumor necrosis factor α
6-OHDA	6-hydroxydopamine
ZO-1	Zonula occludens-1
